# Supplementary material for: Approaching enzymatic catalysis with zeolites or how to select one reaction mechanism competing with others
Source: Nat Commun. 2023 May 19;14:2878. doi: 10.1038/s41467-023-38544-z (PMC10198988; doi:10.1038/s41467-023-38544-z)
Supplement: Supplementary file 1 — Supplementary Information [file 41467_2023_38544_MOESM1_ESM.pdf]

Supplementary Information

Approaching enzymatic catalysis with zeolites or how to select one reaction mechanism competing with others

Pau Ferri, [1†] Chengeng Li,[1†] Daniel Schwalbe-Koda,[2] Mingrou Xie,[3] Manuel Moliner,[1] Rafael Gómez-Bombarelli,[2] Mercedes Boronat,[1*] Avelino Corma[1*]

[1] Instituto de Tecnología Química, Universitat Politècnica de València - Consejo Superior de Investigaciones Científicas, Avenida de los Naranjos s/n, 46022 Valencia, Spain

[2] Department of Materials Science and Engineering, Massachusetts Institute of Technology, Cambridge, MA 02139

[3] Department of Chemical Engineering, Massachusetts Institute of Technology, Cambridge, MA 02139

*Corresponding author: E-mail: acorma@itq.upv.es, boronat@itq.upv.es

† These authors have contributed equally

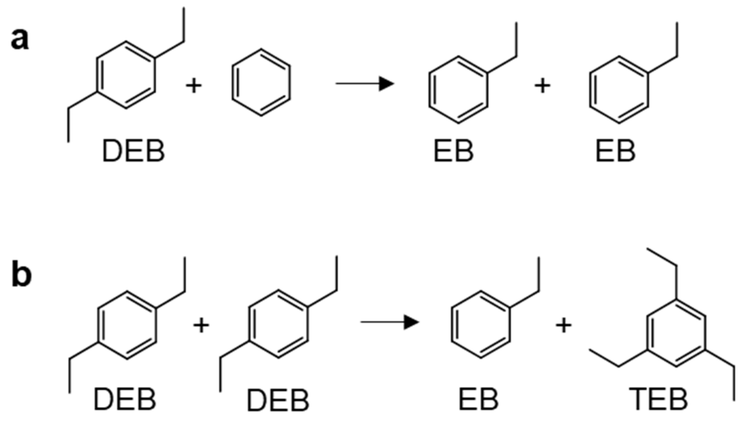

Supplementary Fig. 1. Competitive diethylbenzene reactions. **a** transalkylation with benzene. **b** disproportionation.

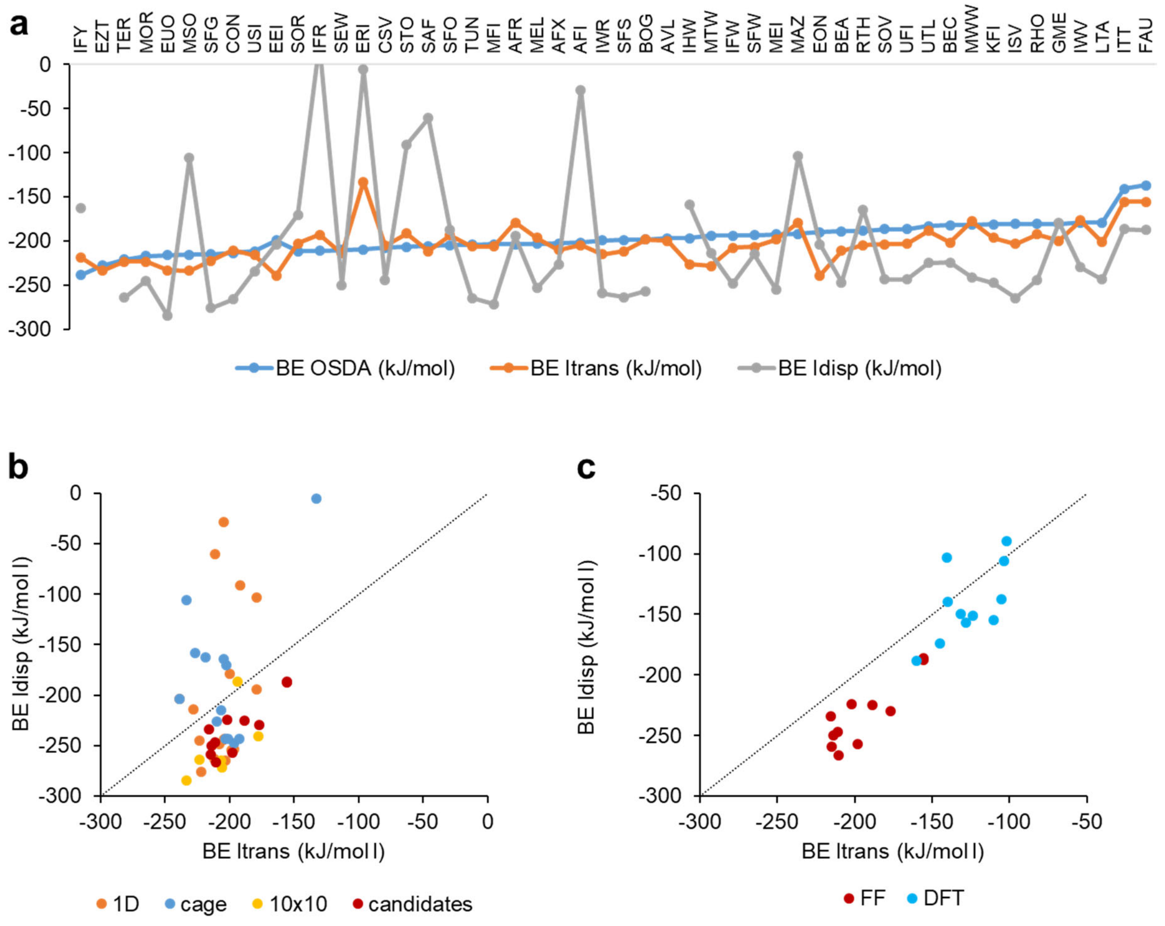

Supplementary Fig. 2. **Results from force fields (FF) simulations. a** Binding energies (BE) of OSDA (blue) and diaryl intermediates for transalkylation (I_{trans}) (orange) and disproportionation (I_{disp}) (gray) from force fields simulations. **b** Correlation between BE I_{trans} and BE I_{disp} for different types of zeolite channels systems calculated with FF. **c** Comparison between FF and DFT for the selected candidates. The dotted lines corresponding to BE I_{disp} = BE I_{trans} are included to guide the eye.

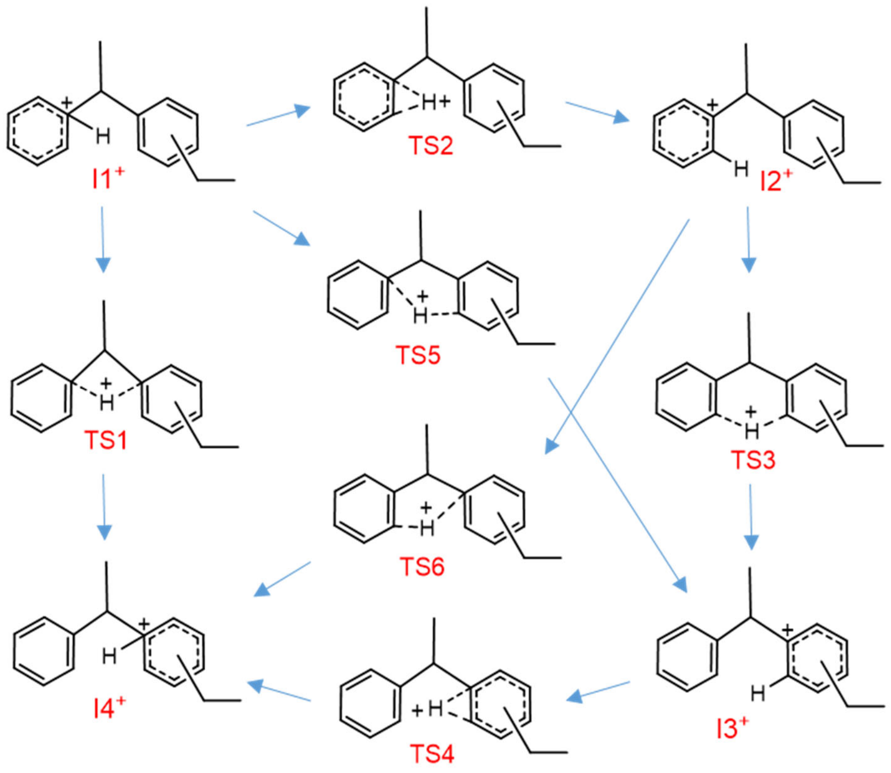

Supplementary Fig. 3 Schematic representation of the diaryl-mediated pathway. Possible pathways for transformation of the cationic diaryl intermediate I1$^+$ into I4$^+$.

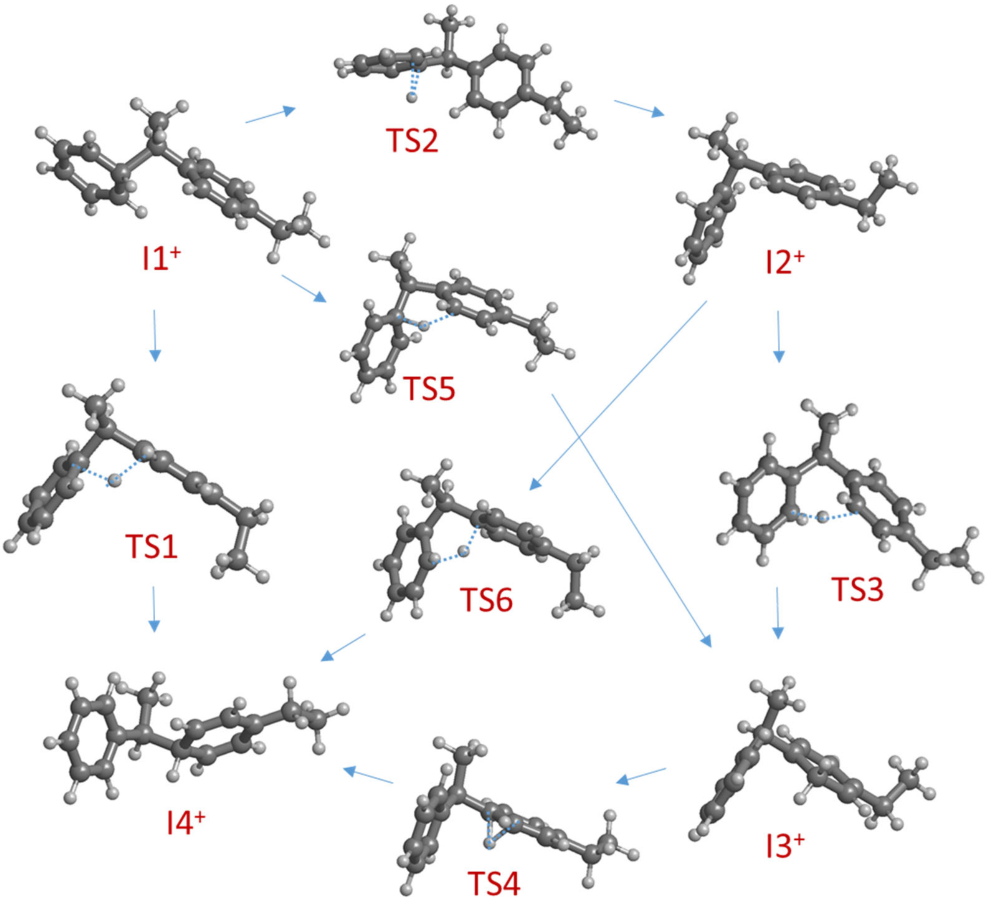

Supplementary Fig. 4. DFT structural results for the diaryl-mediated pathway. Optimized geometries of all minima and transition states involved in the four possible pathways considered for the diaryl-mediated transalkylation of p-DEB$^+$ with benzene in gas phase.

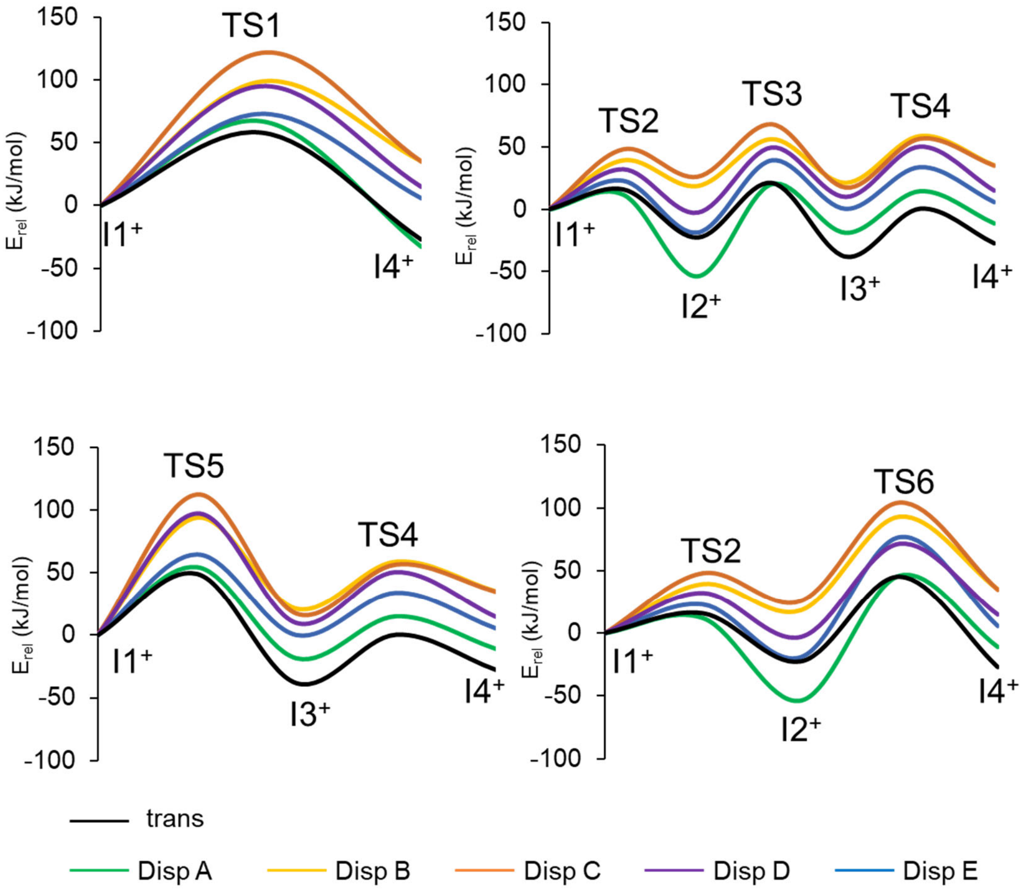

Supplementary Fig. 5. DFT energetic results for the diaryl-mediated pathway. Calculated energy profiles for all transalkylation and disproportionation diaryl-mediated pathways considered in gas phase.

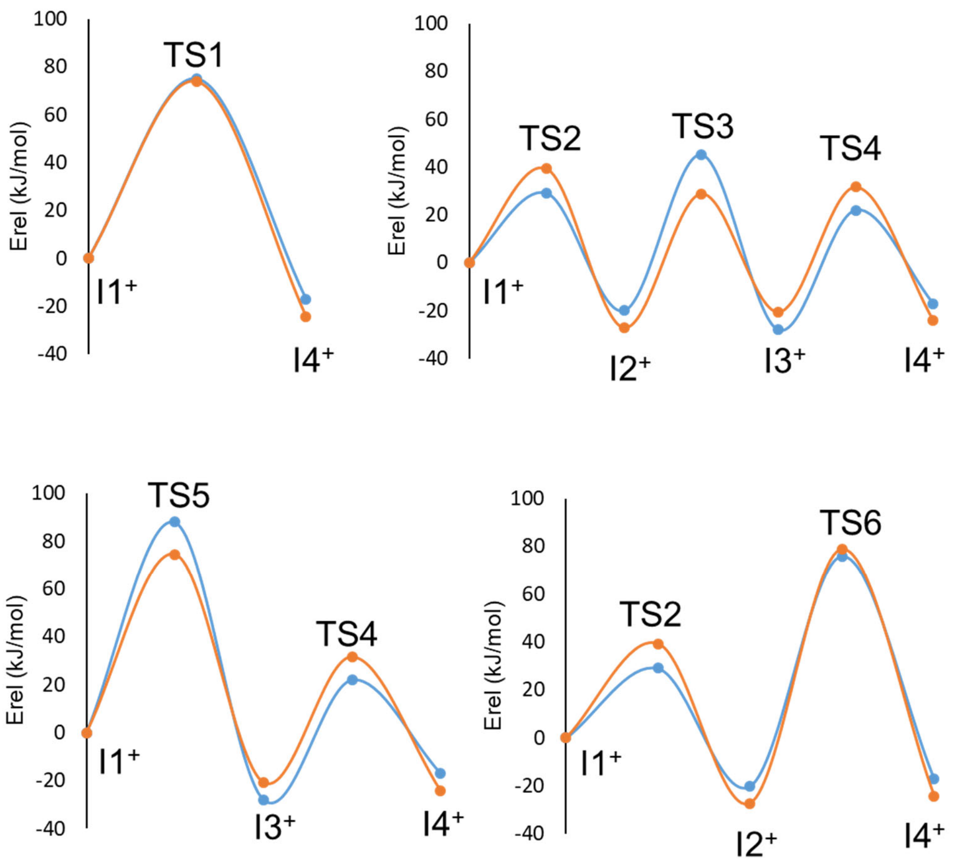

Supplementary Fig. 6. The effect of Al on the diaryl-mediated pathway in BOG. Calculated energy profiles for all transalkylation diaryl-mediated pathways in pure Si (blue) and Al-containing (orange) models of BOG.

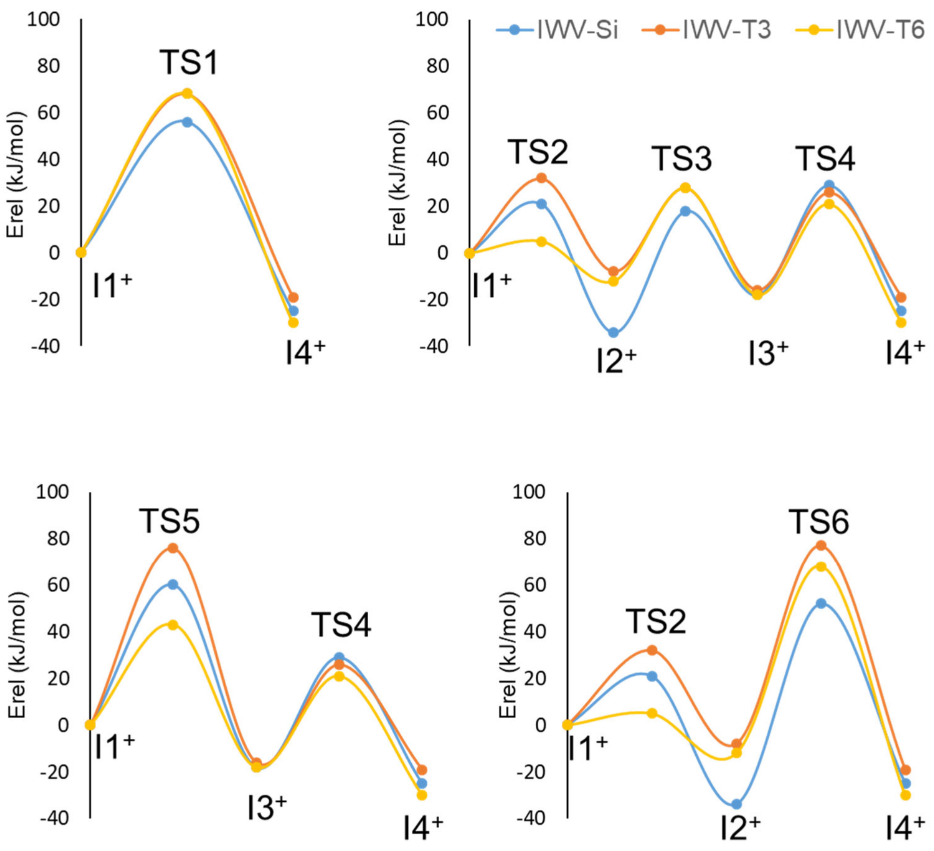

Supplementary Fig. 7. The effect of Al on the diaryl-mediated pathway in IWV. Calculated energy profiles for all transalkylation diaryl-mediated pathways in pure Si (blue) and Al-containing (orange and yellow) models of IWV. Data taken from reference 30.

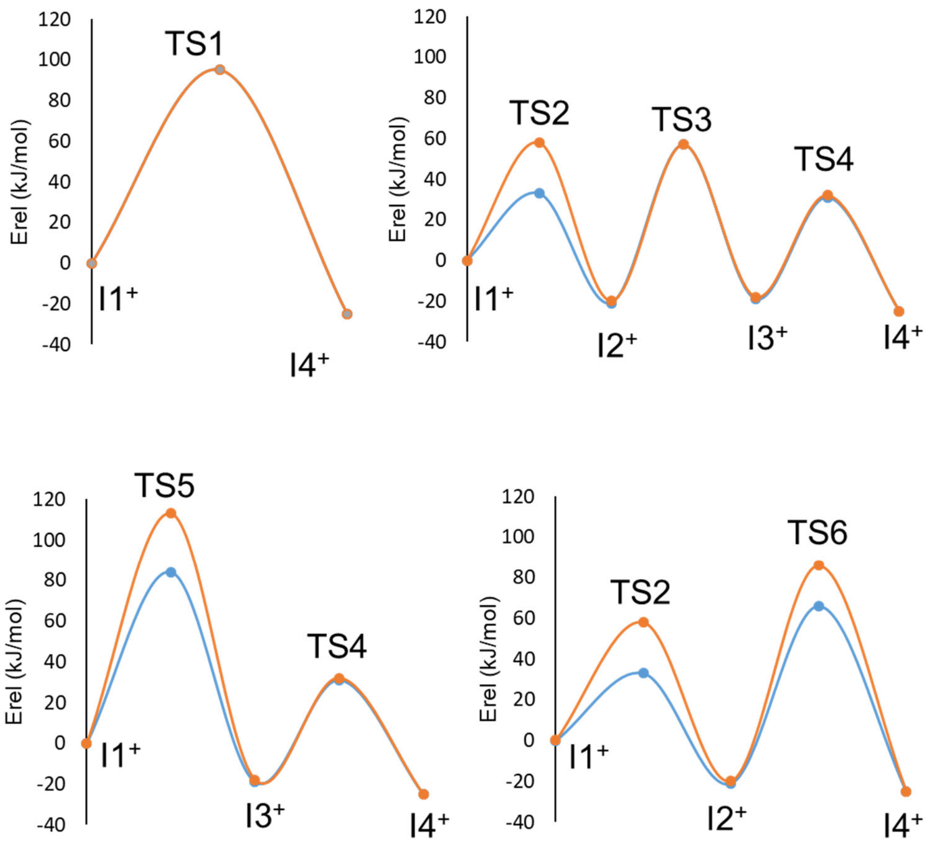

Supplementary Fig. 8. The effect of Al on the diaryl-mediated pathway in MOR. Calculated energy profiles for all transalkylation diaryl-mediated pathways in pure Si (blue) and Al-containing (orange) models of MOR. Data taken from reference 30.

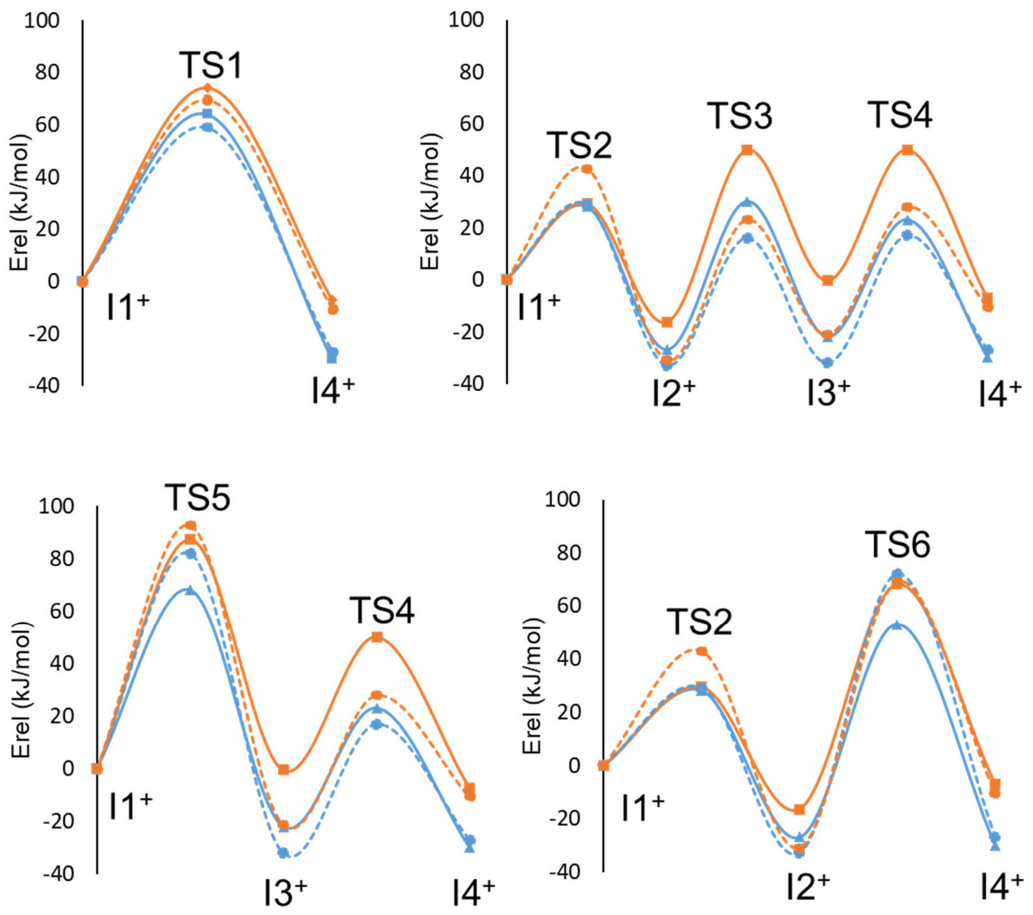

Supplementary Fig. 9. The effect of Al on the diaryl-mediated pathway in UTL. Calculated energy profiles for all transalkylation diaryl-mediated pathways in pure Si (blue) and Al-containing (orange) models of UTL(cha) (full lines) and UTL(int) (dashed lines).

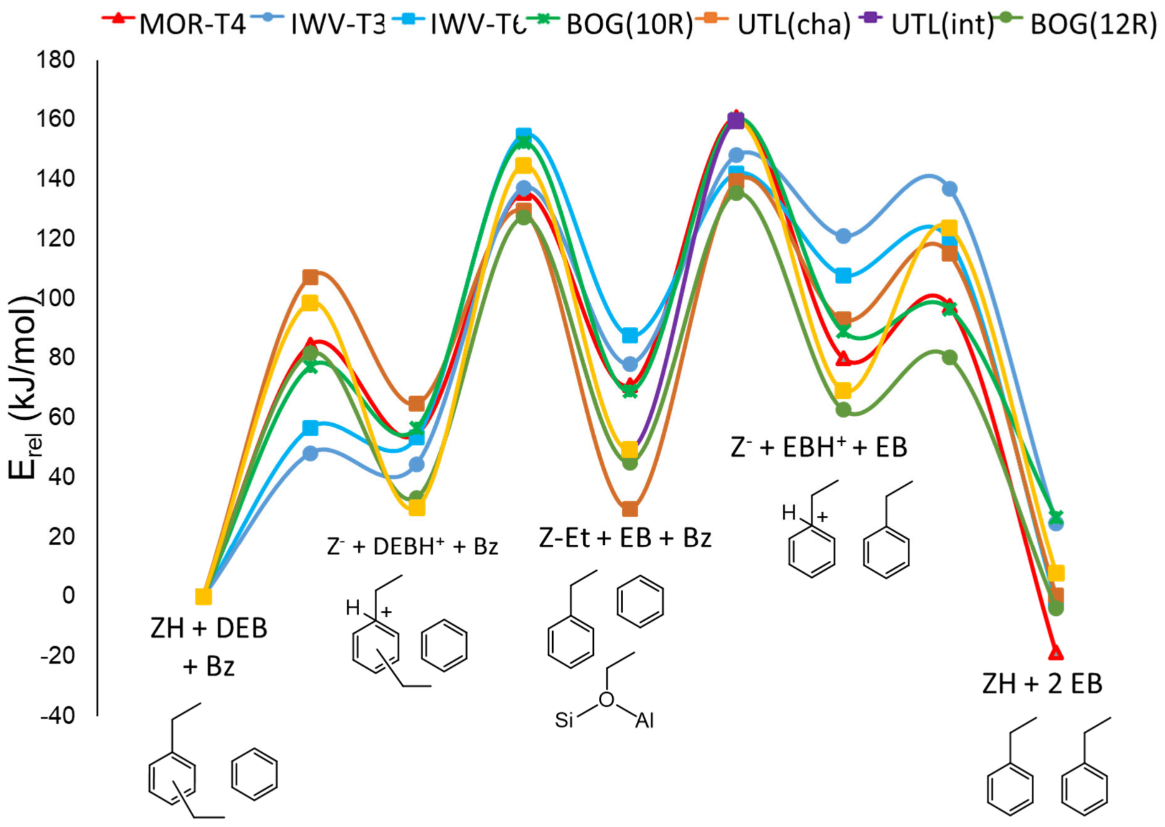

Supplementary Fig. 10. DFT results for the alkyl-transfer pathway. Calculated energy profiles for the alkyl-transfer pathway for transalkylation in BOG, IWV, MOR and UTL zeolite structures. The data for IWV and MOR are taken from reference 30.

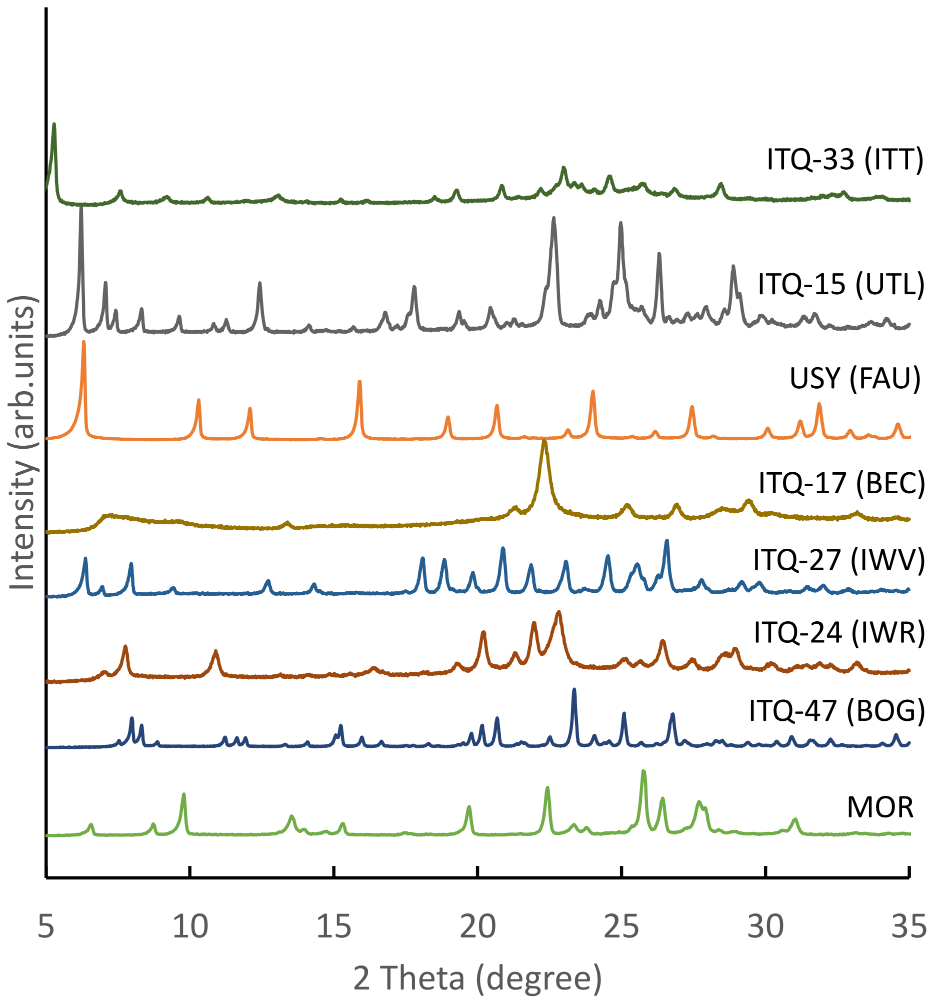

Supplementary Fig. 11. Power X-Ray Diffraction data. PXRD patterns of the zeolites employed in this study

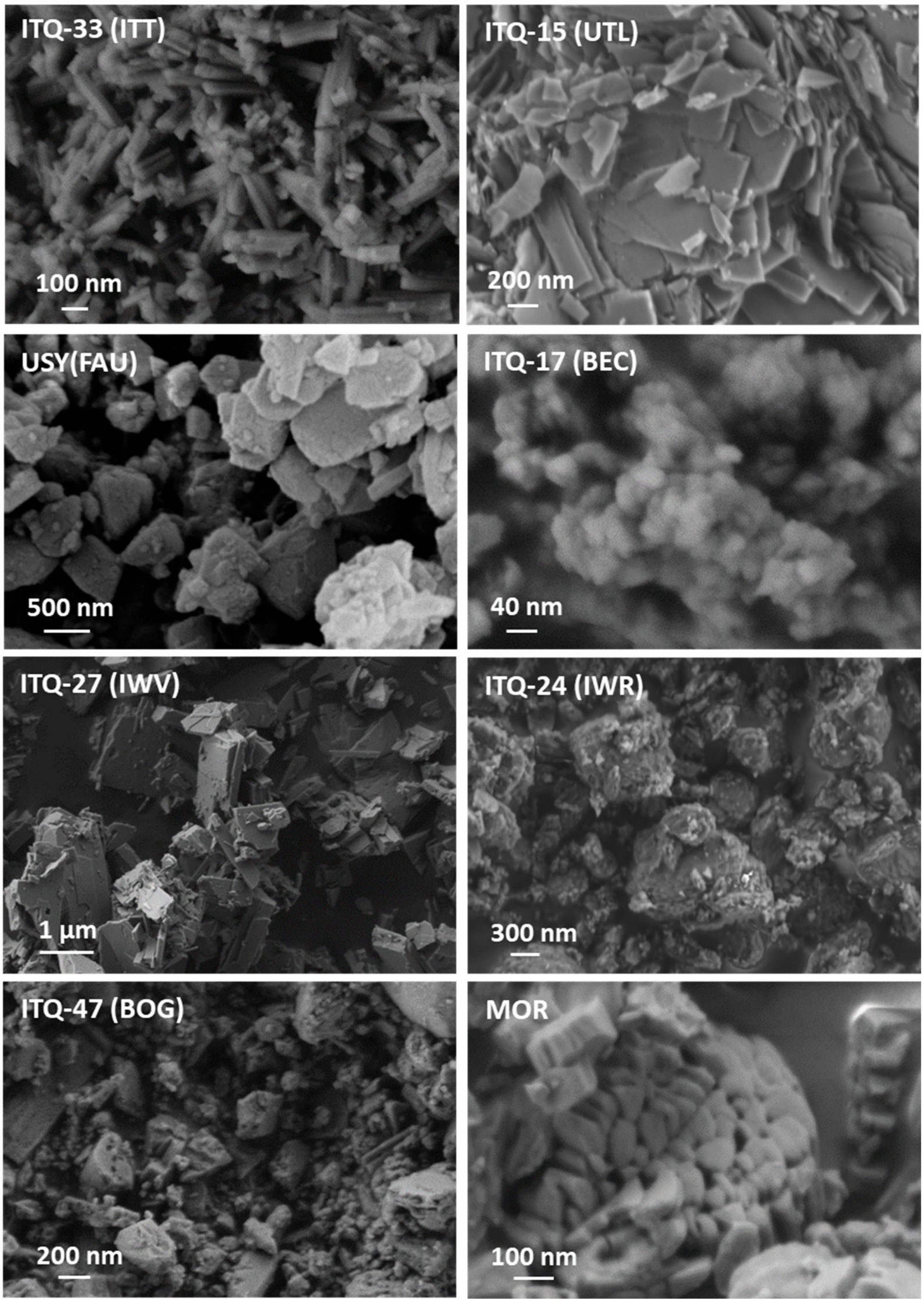

Supplementary Fig. 12. Field emission scanning electron microscopy. FESEM images of zeolite samples employed in this study.

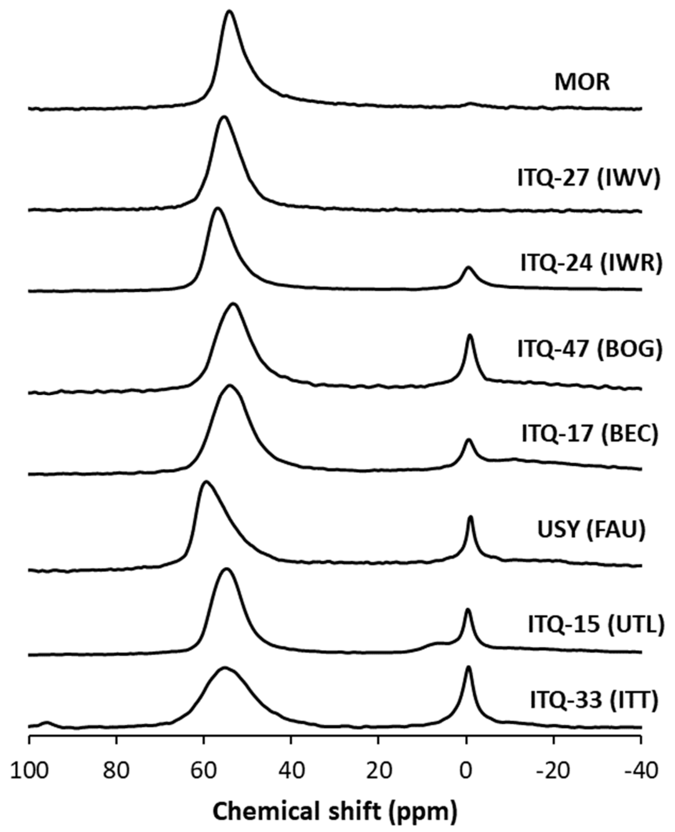

Supplementary Fig. 13. Nuclear Magnetic Resonance characterization. ^{27}Al MAS NMR spectra of the zeolite catalysts employed in this study.

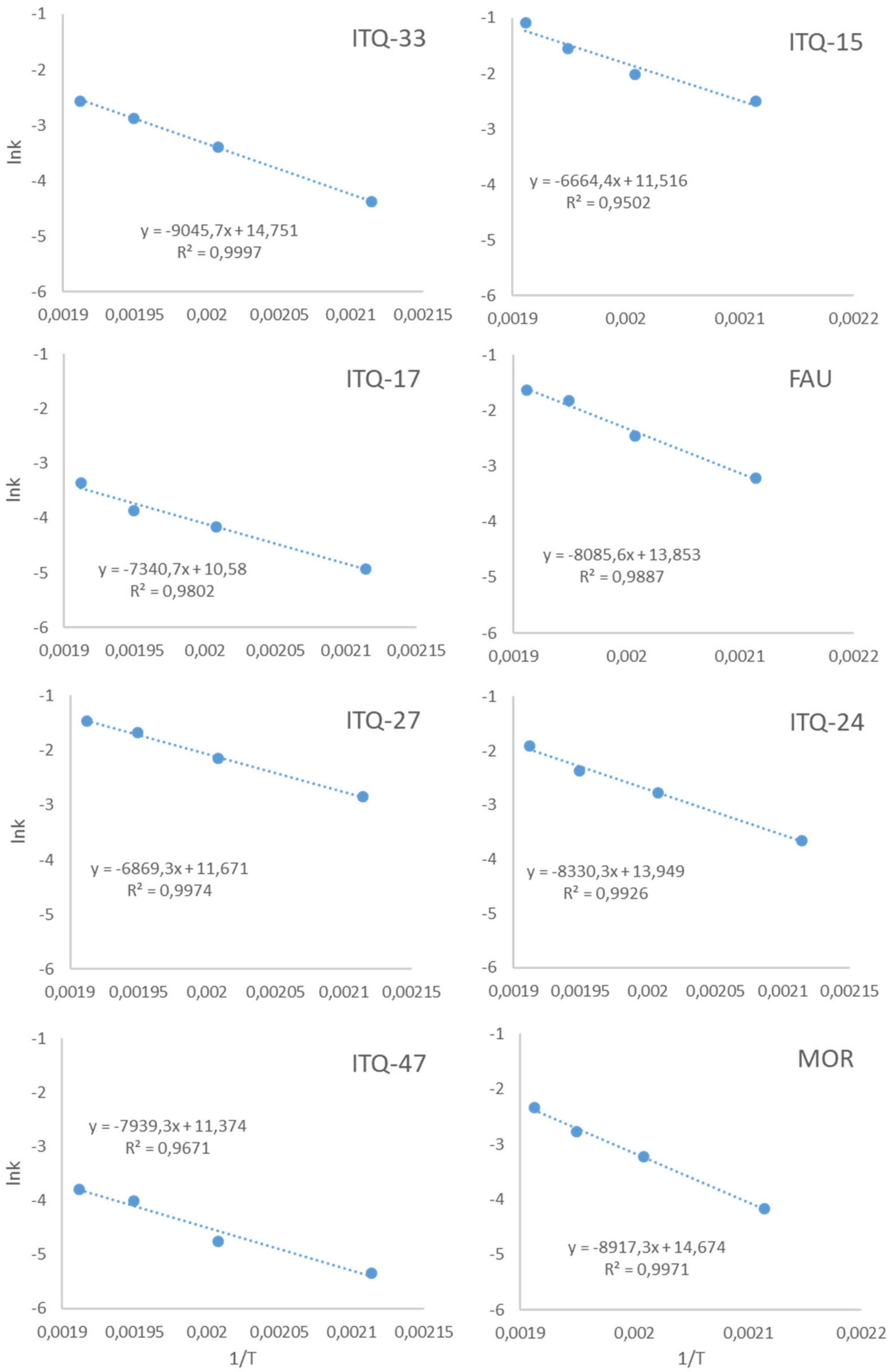

Supplementary Fig. 14. Kinetic Study and activation energies. Plots of ln*k* versus 1/T for benzene-diethylbenzene transalkylation on different zeolite structures. The reaction rate constants (*k*) and T values are summarized in Supplementary Table 12.

Supplementary Table 1. Binding energies of OSDA and key intermediates for transalkylation (I_{trans}) and disproportionation (I_{disp}) from FF simulations.

IZA code	Channels System	BE OSDA (kJ/mol)	BE I_{trans} (kJ/mol)	BE I_{disp} (kJ/mol)
AFI	12	-202	-205	-29
AFR	12x8	-203	-179	-194
AFX	8	-203	-210	-226
AVL	8	-197	-200	-
BEA	12x12	-189	-211	-247
BEC	12x12x12	-182	-202	-224
BOG	12x10	-198	-198	-257
CON	12x10x10	-213	-210	-266
CSV	10x8	-207	-205	-244
EEI	8	-199	-239	-204
EON	12x8x8	-190	-239	-204
ERI	8	-209	-133	-6
EUO	10x10	-216	-233	-285
EZT	12	-228	-234	-
FAU	12x12x12	-137	-156	-187
GME	12x8	-180	-200	-179
IFR	12	-211	-193	24
IFW	10x8x8	-194	-208	-248
IFY	8	-238	-218	-163
IHW	8	-197	-226	-158
ISV	12	-181	-203	-265
ITT	18x10x10	-141	-156	-186
IWR	12x10x10	-199	-215	-259
IWV	12x12	-179	-177	-230
KFI	8	-181	-196	-247
LTA	8	-179	-201	-243

MAZ	12x8x8	-192	-179	-104
MEI	12	-192	-198	-255
MEL	10	-203	-196	-253
MFI	10x10	-203	-206	-271
MOR	12x8	-217	-223	-245
MSO	6	-215	-233	-106
MTW	12	-194	-228	-214
MWW	10x10	-181	-178	-241
RHO	8	-180	-192	-244
RTH	8	-188	-204	-164
SAF	12	-206	-211	-61
SEW	12x10	-210	-214	-250
SFG	10	-215	-222	-276
SFO	10x10	-204	-194	-187
SFS	12x8	-198	-212	-263
SFW	8	-194	-207	-215
SOR	8	-212	-203	-170
SOV	12x8	-186	-204	-244
STO	12	-207	-192	-91
TER	10x10	-221	-223	-264
TUN	10x10x10	-204	-206	-265
UFI	8	-186	-203	-244
USI	12x10	-212	-216	-234
UTL	14x12	-183	-188	-225

Supplementary Table 2. Relative stability of ortho-, meta- and para-isomers of neutral DEB, DEB^+ carbenium ion, and cationic diaryl intermediates involved in transalkylation I_{trans} and disproportionation I_{disp} reactions, in kJ/mol.

Isomers of neutral diethylbenzene (DEB)		
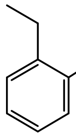	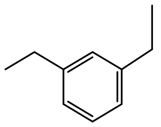	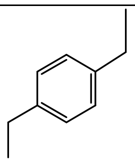
o-DEB<br>$E_{rel} = 2.1$	m-DEB<br>$E_{rel} = 0.0$	p-DEB<br>$E_{rel} = 0.4$
Isomers of diethylbenzenium cation (DEB^+)		
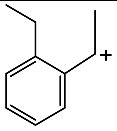	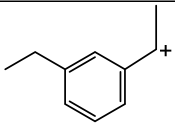	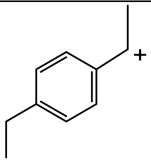
o-DEB^+<br>$E_{rel} = 14.2$	m-DEB^+<br>$E_{rel} = 15.4$	p-DEB^+<br>$E_{rel} = 0.0$
Diaryl intermediates		
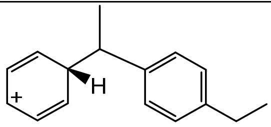	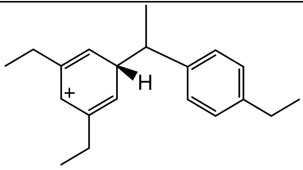	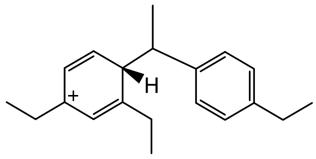
I_{trans}	I_{disp} A<br>$E_{rel} = 0.0$	I_{disp} B<br>$E_{rel} = 3.8$
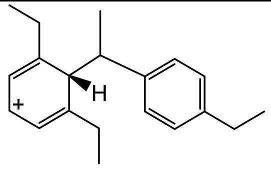	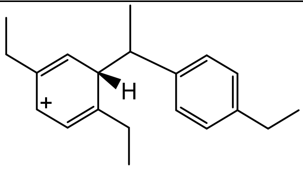	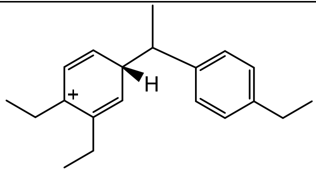
I_{disp} C<br>$E_{rel} = 3.1$	I_{disp} D<br>$E_{rel} = 4.0$	I_{disp} E<br>$E_{rel} = 10.4$

Supplementary Table 3. Relative stability (in kJ/mol) of intermediates and transition states involved in the mechanisms of transalkylation and disproportionation through the different diaryl isomers depicted in Supplementary Table 2 in gas phase.

	transalkylation	disproportionation				
		A	B	C	D	E
$I1^+$	0	0	0	0	0	0
TS1	58	67	99	121	95	73
$I4^+$	-27	-11	35	35	15	6
TS2	16	12	39	48	32	23
$I2^+$	-23	-54	19	26	-3	-20
TS3	21	20	57	68	50	40
$I3^+$	-38	-19	21	17	10	0
TS4	0	15	59	57	51	34
TS5	49	54	94	113	97	65
TS6	45	46	93	105	72	77

Supplementary Table 4. Calculated activation barriers (in kJ/mol) for all the elementary steps of the mechanisms of transalkylation and disproportionation through the different diaryl isomers depicted in Supplementary Table 2 in gas phase.

	transalkylation	disproportionation				
		A	B	C	D	E
Ea1	58	67	99	121	95	73
Ea2	16	12	44	62	32	23
Ea3	44	74	61	47	53	58
Ea4	38	33	45	52	41	34
Ea5	49	54	94	113	97	65
Ea6	68	100	96	77	75	95

Supplementary Table 5. Relative stability of Al location in BOG and UTL zeolite structures.

BOG	Erel (kJ/mol)	UTL	Erel (kJ/mol)
T1	0	T1	35
T2	23	T2	41
T3	35	T3	16
T4	24	T4	11
T5	6	T5	28
T6	6	T6	39
		T7	37
		T8	20
		T9	19

Supplementary Table 6. Relative stability (in kJ/mol) of intermediates and transition states involved in the mechanisms of transalkylation in pure silica and Al-containing models of BOG, IWV, MOR and UTL zeolite structures. The data for IWV and MOR are taken from reference 30.

	BOG		IWV			MOR		UTL (int)		UTL (cha)	
	Si	Al-T1	Si	Al-T3	Al-T6	Si	Al-T4	Si	Al-T11	Si	Al-T11
$I1^+$	0	0	0	0	0	0	0	0	0	0	0
TS1	75	74	56	68	68	95	95	59	70	64	74
$I4^+$	-17	-24	-25	-19	-30	-25	-25	-27	-11	-30	-7
TS2	29	39	21	32	5	33	58	29	43	28	29
$I2^+$	-20	-27	-34	-8	-12	-21	-20	-33	-31	-27	-16
TS3	45	29	18	28	28	57	57	16	23	30	50
$I3^+$	-28	-21	-18	-16	-18	-19	-18	-32	-21	-22	0
TS4	22	32	29	26	21	31	32	17	28	23	50
TS5	88	74	60	76	43	84	113	82	93	68	93
TS6	76	79	52	77	68	66	86	72	69	53	68

Supplementary Table 7. Relative stability (in kJ/mol) of intermediates and transition states involved in the diaryl-mediated mechanisms of transalkylation and disproportionation in pure silica models of different zeolite structures.

	transalkylation							disproportionation			
	BEC	BOG	IWR	IWV	MOR	UTL (int)	UTL (cha)	BEC	IWV	UTL (int)	UTL (cha)
$I1^+$	0	0	0	0	0	0	0	0	0	0	0
TS1	98	75	71	56	95	59	64	113	72	90	80
$I4^+$	-32	-17	-19	-25	-25	-27	-30	-17	-4	12	-11
TS2	35	29	31	21	33	29	28	30	30	29	22
$I2^+$	-29	-20	-24	-34	-21	-33	-27	-29	-60	-47	-45
TS3	15	45	36	18	57	16	30	27	15	22	28
$I3^+$	-29	-28	-30	-18	-19	-32	-22	-9	-14	-10	2
TS4	20	22	14	29	31	17	23	46	45	48	48
TS5	85	88	91	60	84	82	68	78	67	71	82
TS6	69	76	66	52	66	72	53	89	44	51	52

Supplementary Table 8. Optimized values of the α, β, γ and δ angles described in Fig. 5.

		gas	BEC	BOG	IWR	IWV	MOR	UTL(int)	UTL(cha)
$I1^{+}$	α	108	110	108	108	108	112	109	112
	β	128	130	124	128	121	132	123	126
	γ	118	132	124	123	121	142	133	150
	δ	178	174	178	176	179	171	178	176
TS1	α	101	105	97	97	99	101	100	98
	β	98	118	94	104	98	113	100	97
	γ	180	172	179	176	180	170	178	178
	δ	178	167	175	178	178	172	180	179
$I2^{+}$	α	104	109	109	103	101	115	112	100
	β	100	113	114	102	97	122	109	94
	γ	175	179	177	177	175	173	176	173
	δ	178	174	175	179	178	173	175	178
TS3	α	106	107	109	107	107	109	107	107
	β	96	97	101	97	97	102	98	96
	γ	171	171	169	171	170	175	173	170
	δ	172	171	177	172	172	171	168	172
$I3^{+}$	α	110	108	116	104	110	115	109	112
	β	109	106	118	104	106	119	110	111
	γ	179	178	177	179	174	176	177	176
	δ	176	175	176	178	179	173	178	176
$I4^{+}$	α	109	111	110	109	108	113	111	109
	β	123	124	124	122	118	135	129	125
	γ	179	179	177	179	176	176	177	178
	δ	128	135	135	151	138	129	140	131
TS5	α	103	104	106	105	105	107	101	106
	β	96	104	108	99	97	109	100	106
	γ	175	171	170	179	179	172	166	173
	δ	166	173	173	171	167	159	167	175
TS6	α	110	105	108	100	102	108	104	102
	β	109	111	102	90	94	112	99	95
	γ	179	166	163	163	164	163	166	163
	δ	176	158	175	179	173	165	173	175

Supplementary Table 9. Relative stability (in kJ/mol) of intermediates and transition states involved in the mechanisms of transalkylation in pure silica and Al-containing models of BOG, IWV, MOR and UTL zeolite structures. The data for IWV and MOR are taken from reference 30.

	BOG		IWV		MOR	UTL	
	10R	12R	T3	T6	T4	12R	14R
ZH+ DEB + Bz	0	0	0	0	0	0	0
TS7	77	82	48	57	84	99	107
Z^- + $DEBH^+$ +Bz	56	33	44	53	54	30	65
TS8	152	127	137	155	135	145	129
Z-Et + EB + Bz	69	45	78	88	71	49	30
TS9	160	135	148	142	161	159	142
Z^- + EBH^+ +EB	89	63	121	108	80	69	93
TS10	97	80	137	120	98	124	115
ZH + 2 EB	27	-4	25	-2	-19	8	0

Supplementary Table 10. Physicochemical properties of zeolite materials employed in this study.

zeolite	IZA code	(Si+Ge) /Al	Si/Ge	S_{BET} (m^2/g)	V_{micro} (cm^3/g)	S_{ext} (m^2/g)	Acid amount (µmol/g)	
							150ºC	350ºC
ITQ-33	ITT	19.1	2.3	650	0.28	60	278	150
ITQ-15	UTL	10	49.7	470	0.21	33	434	228
USY	FAU	13.1		723	0.32	75	478	296
ITQ-17	BEC	10.8	3.4	374	0.13	102	348	116
ITQ-27	IWV	23.8		460	0.22	20	309	175
ITQ-24	IWR	13.5		346	0.15	45	389	257
ITQ-47	BOG	24.3	85.5	147	0.18	21	417	147
Mordenite	MOR	12.7		406	0.19	11	733	361

Supplementary Table 11. Results of catalytic test in diethylbenzene-benzene transalkylation at 240ºC.

zeolite	IZA code	w/F (h)	DEB conv. (%)	r_{trans} (mol_{EB}/ mol_{acid}h)	EB (%)	TEB (%)	C_2H_4 (%)	HP (%)
ITQ-33	ITT	0.84	11.9	717	76.4	20.8	0.6	2.1
ITQ-15	UTL	0.42	17.7	1599	85.7	12.0	0	2.3
USY	FAU	0.42	14.7	1075	90.1	2.7	2.7	4.5
ITQ-17	BEC	2.53	20.8	628	88.8	8.3	0.5	2.3
ITQ-27	IWV	0.42	14.9	1926	94.1	0.5	2.3	3.0
ITQ-24	IWR	0.84	15.2	676	95.1	2.5	0.4	2.0
ITQ-47	BOG	2.53	14.4	349	90.4	4.4	4.8	0.4
mordenite	MOR	0.84	9.6	279	88.6	2.5	4.6	4.2

Supplementary Table 12. Reaction rate constant (*k*) of benzene-diethylbenzene transalkylation at EB yield below 20%. Reaction conditions: Pressure: 1 atm, feeding: N_2:DEB:Bz = 30:1:5 (mol).

zeolite	IZA code	T (K)			
		473	498	513	523
ITQ-33	ITT	0.0125	0.0334	0.0565	0.0773
ITQ-15	UTL	0.0829	0.134	0.211	0.338
USY	FAU	0.04	0.0849	0.163	0.194
ITQ-17	BEC	0.0073	0.0157	0.0212	0.0348
ITQ-27	IWV	0.0583	0.1157	0.1861	0.2286
ITQ-24	IWR	0.026	0.0626	0.093	0.1478
ITQ-47	BOG	0.0048	0.0086	0.0182	0.0227
Mordenite	MOR	0.0154	0.04	0.0628	0.0969

Supplementary Table 13. Crystallographic description of the zeolite frameworks.

IZA code	Cell Type	Unit Cell Parameters						T^a
		a (Å)	b (Å)	c (Å)	α (º)	β (º)	γ (º)	
BEA	Tetragonal	12.593	12.593	26.495	90	90	90	64
BEC	Tetragonal	12.935	12.822	12.676	90	90	90	32
BOG-F	Orthorhombic	20.104	23.659	12.799	90	90	90	96
BOG-R	Triclinic	12.812	16.791	16.791	74	68	68	48
CON	Monoclinic	22.459	13.613	12.409	90	70	90	56
FAU	Cubic	24.345	24.345	24.345	90	90	90	192
ITT	Hexagonal	19.039	19.027	11.512	90	90	120	46
IWR	Orthorhombic	21.112	13.570	12.608	90	90	90	56
IWV	Triclinic	14.544	15.784	19.055	48	54	78	38
MOR	Orthorhombic	18.023	20.042	7.432	90	90	90	48
SEW	Orthorhombic	24.217	11.422	14.518	90	90	90	132
USI	Monoclinic	21.209	13.001	19.338	90	109	90	40
UTL-F	Monoclinic	29.160	14.098	12.180	90	105	90	76
UTL-R	Triclinic	12.446	13.962	16.199	65	76	90	38

[a]Number of T atoms in the unit cell.
